# Integration of hybridization-based markers (overgos) into physical maps for comparative and evolutionary explorations in the genus *Oryza *and in *Sorghum*

**DOI:** 10.1186/1471-2164-7-199

**Published:** 2006-08-08

**Authors:** Barbara L Hass-Jacobus, Montona Futrell-Griggs, Brian Abernathy, Rick Westerman, Jose-Luis Goicoechea, Joshua Stein, Patricia Klein, Bonnie Hurwitz, Bin Zhou, Fariborz Rakhshan, Abhijit Sanyal, Navdeep Gill, Jer-Young Lin, Jason G Walling, Mei Zhong Luo, Jetty Siva S Ammiraju, Dave Kudrna, Hye Ran Kim, Doreen Ware, Rod A Wing, Phillip San Miguel, Scott A Jackson

**Affiliations:** 1 Department of Agronomy, Purdue University, West Lafayette, Indiana 47907, USA; 2 Department of Horticulture, Purdue University, West Lafayette, Indiana 47907, USA; 3 Arizona Genomics Institute, University of Arizona, Tucson, Arizona 85721, USA; 4 Cold Spring Harbor Laboratory, Cold Spring Harbor, New York 11724, USA; 5 USDA-ARS NAA Plant, Soil & Nutrition Laboratory Research Unit, Ithaca, New York 14853, USA; 6 The Institute for Plant Genomics and Biotechnology, Texas A&M University, College Station, Texas 77843, USA; 7 Present address: Microarray Shared Resource-AGTC, Mayo Clinic, Rochester, MN 55905, USA

## Abstract

**Background:**

With the completion of the genome sequence for rice (*Oryza sativa *L.), the focus of rice genomics research has shifted to the comparison of the rice genome with genomes of other species for gene cloning, breeding, and evolutionary studies. The genus *Oryza *includes 23 species that shared a common ancestor 8–10 million years ago making this an ideal model for investigations into the processes underlying domestication, as many of the *Oryza *species are still undergoing domestication. This study integrates high-throughput, hybridization-based markers with BAC end sequence and fingerprint data to construct physical maps of rice chromosome 1 orthologues in two wild *Oryza *species. Similar studies were undertaken in *Sorghum bicolor*, a species which diverged from cultivated rice 40–50 million years ago.

**Results:**

Overgo markers, in conjunction with fingerprint and BAC end sequence data, were used to build sequence-ready BAC contigs for two wild *Oryza *species. The markers drove contig merges to construct physical maps syntenic to rice chromosome 1 in the wild species and provided evidence for at least one rearrangement on chromosome 1 of the *O. sativa *versus *Oryza officinalis *comparative map. When rice overgos were aligned to available *S. bicolor *sequence, 29% of the overgos aligned with three or fewer mismatches; of these, 41% gave positive hybridization signals. Overgo hybridization patterns supported colinearity of loci in regions of sorghum chromosome 3 and rice chromosome 1 and suggested that a possible genomic inversion occurred in this syntenic region in one of the two genomes after the divergence of *S. bicolor *and *O. sativa*.

**Conclusion:**

The results of this study emphasize the importance of identifying conserved sequences in the reference sequence when designing overgo probes in order for those probes to hybridize successfully in distantly related species. As interspecific markers, overgos can be used successfully to construct physical maps in species which diverged less than 8 million years ago, and can be used in a more limited fashion to examine colinearity among species which diverged as much as 40 million years ago. Additionally, overgos are able to provide evidence of genomic rearrangements in comparative physical mapping studies.

## Background

Rice is the world's most important agronomic plant. In Asia, home to 70% of the world's poor, rice provides up to two-thirds of the population's daily caloric intake and as much as 60% of the daily protein intake [1]. Efforts to improve our understanding of rice genetics, thereby improving the breeding and cultivation of rice, have led to numerous international collaborations, culminating in the completion of a publicly available genome sequence of rice in 2002 [2,3]. This sequence provides a powerful tool for the identification of agronomically important genes in rice, as well as for the identification of orthologous regions in other crop species. With a sequence in hand, the focus for rice geneticists has shifted to the integration of rice physical and genetic maps[4], and comparison of the rice genome to genomes of other species, both for comparative gene cloning efforts and evolutionary studies [5,6].

The genus *Oryza*, of which cultivated rice (*Oryza sativa *L.) is a member, includes 23 species from a widely diverse range of habitats. Wild rice species can be found in Central and South America, Australia, Africa, and Asia, while *O. sativa *is distributed worldwide, in both temperate and tropical climates [7]. Rice research is now looking to the wild rice species to help answer questions about domestication, speciation, polyploidy, and to furnish genes for breeding purposes [8]. However, there are no plans at this time for an international full genome sequencing effort of these species. Therefore, other methods must be employed to harness the knowledge hidden in these wild rice genomes.

Recent comparative genomics studies in animals have used overgo (overlapping oligonucleotide) probes to construct sequence-ready contigs of large-insert clones in multiple species. Thomas et al. (2002) [9] designed probes from conserved regions of human-mouse alignments and used them to construct sequence-ready, bacterial artificial chromosome (BAC)-based physical maps for six other mammalian species. More recently, Kellner et al. (2005) [10] reported improved probe design algorithms that are being used to design overgo probe sets which can identify most genes or regions of interest in placental mammals, birds, and reptiles. These probes are again constructed using whole genome alignments between human, mouse, and rat as the basis for probe design. In order to build BAC-based physical maps of wild rice, we used the rice sequence to design overgo probes for hybridization to BAC libraries of eleven wild rice species. Because no other monocot species has been completely sequenced, we could not use multiple-species sequence alignments for probe design, and instead relied on hits to plant EST databases to design probes from putatively conserved regions.

**Figure 1:**
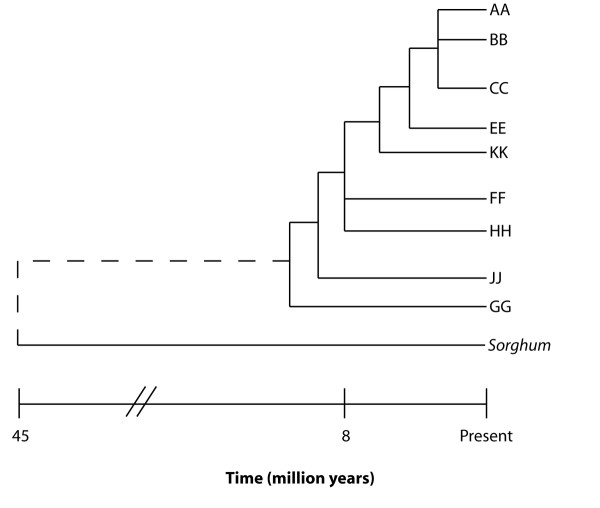
**Phylogeny of *Oryza *and *Sorghum***. Simplified phylogeny showing the estimated divergence times of *Sorghum *and *Oryza *species. *Oryza *lineages are summarized by genome type, designated AA through JJ according to morphological, physiological, biochemical, and molecular differences, including chromosome pairing behavior of F_1 _hybrids from interspecific crosses [45]. This diagram is based on the data of Gaut et al. (2002) [13] and Ge et al. (1999) [11].

Cultivated rice is a diploid species (2n = 24) of the AA genome type [7,8,11]. The wild rice species used in this study represent a diverse evolutionary background, including both diploid and tetraploid species encompassing 8–10 million years of divergence [12] (Figure 1). In addition to the *Oryza *species, we tested the effectiveness of rice overgos when hybridized to *Sorghum bicolor *L. Moench, a species within the Gramineae but outside of the genus *Oryza *that diverged from rice 40–50 million years ago [13] (Figure 1).

We will discuss the success rates of our overgos in hybridizations to *O. sativa*, the species from which the overgos were designed, to eleven wild rice species in the genus *Oryza*, and to the more distantly related grass *S. bicolor*. We will then report on the use of these overgos for ongoing physical and comparative mapping projects in two of the wild rice species and for comparative mapping in *S. bicolor*.

## Results

### Overgo design and hybridization success in *Oryza *species

The goal of this project was to design overgo markers from the rice (*O. sativa*) genomic sequence [14], hybridize those overgos to BAC libraries constructed from wild *Oryza *species, and use the data generated, in conjunction with BAC fingerprinting and BAC end sequencing data generated by the *Oryza *Map Alignment Project (OMAP) [15], to link the physical maps of the wild *Oryza *species to the *O. sativa *reference genome sequence. The first step toward achieving this goal was to identify potential overgos from the rice reference sequence. In total, 1728 of the potential overgo marker sequences spanning all sequence contigs of *O. sativa *chromosome 1, were identified as having fit our selection criteria. While our optimum target density was one overgo every 25,000 bp along rice chromosome 1, the spacing between selected overgos along the chromosome ranged from 20,001 bp apart to 58,526 bp apart, for a mean spacing of 24,401 bp between overgos (Table 1). To test the efficacy of overgos designed from sequences of other rice chromosomes, an additional 144 overgos were designed from chromosome 3 and were spaced an average of 246,268 bp apart (Table 1). 864 of the selected overgos (6 12 × 12 two dimensional pooling arrays) were used for BAC library hybridizations. Data from 72 of the 864 overgos hybridized, whose failures were traced to laboratory errors, were not included in the further analyses.

**Table 1:** Spacing between overgos designed from rice chromosomes 1 and 3

			Spacing Between Overgos (bp)					
					
Chr. 1 Contig	Contig Size (kb)	No. Overgos Designed	Mean	Median	Min	Max	No. Overgos >40 kb Apart	No. Overgos >50 kb Apart
1	10048	410	24509	22952	20004	48759	4	0
2	1404	60	23411	21394	20021	50917	1	1
3	3429	141	24319	22012	20007	50511	3	1
4	1302	54	24121	22814	20001	34254	0	0
5	1732	72	24065	22596	20001	42902	1	0
6	6676	272	24545	22788	20007	46403	4	0
7	13884	565	24575	22993	20006	58526	6	2
8	810	35	23146	21586	20003	31792	0	0
9	2876	119	24172	22807	20004	40488	1	0
Chr. 1 Total		1728	24401	ND	20021	58526	20	4
Chr. 3 Total		144	246268	58528	24407	4265522	124	109

**Figure 2:**
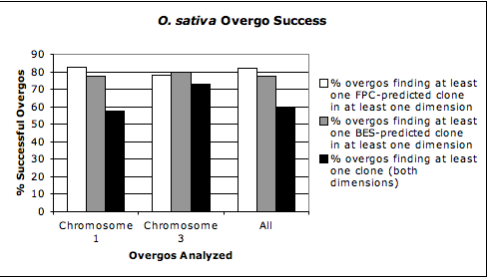
***O. sativa *overgo success**. Percentage of overgos estimated to have been successful in one dimension, based on FPC and BES maps, and the percentage of overgos that were successful in both dimensions. All overgos were designed from the *O. sativa *sequence and hybridized in 12-overgo pools to an *O. sativa *BAC library.

We labelled an overgo as successful if the overgo identified at least one positive clone. Figure 2 shows the success rates of overgos hybridized to the *O. sativa *library as determined by three methods. The first two analyses shown on the chart were performed to provide an estimate of the success of overgos in one dimension when used as pooled probes. Previous studies have defined overgo success as an overgo identifying at least one positive clone, regardless of whether the clone actually hybridized to the sequence from which it was designed. In addition to doing the same analysis in our study, we desired a more accurate picture of overgo success in terms of whether overgos are actually hybridizing to the sequences in the regions from which they were designed. While some clone data in FPC and BES alignments to the rice sequence data is ambiguous, these alignments can provide an estimate of overgo success, defined as an overgo successfully hybridizing to the clone(s) to which we expect it to hybridize. Predicted clone data from the alignments of the FPC and BES maps to the rice pseudomolecule were used to calculate the percentage of overgos that identified at least one FPC or BES clone, respectively, in at least one dimension. The list of BAC clones identified by a twelve-overgo pool was compared to a list of clones expected to be detected by a given overgo from that pool, based on either FPC or BES data. If at least one predicted clone was found by the pool, the overgo was counted as successful. These two analyses can only be used for *O. sativa*, the positive control species, because that is the only species for which we have complete, sequence-anchored fingerprint (FPC) and BES data. Overall, 82% of the 792 overgos tested found at least one FPC-predicted clone in at least one dimension, while 78% of the overgos found at least one BES-predicted clone in at least one dimension. Broken down by chromosome, 83% of the chromosome 1 overgos tested found at least one FPC-predicted clone in at least one dimension, while 77% of the overgos found at least one BES-predicted clone in at least one dimension. 78% of the chromosome 3 overgos tested found at least one FPC-predicted clone in at least one dimension, while 80% of the overgos found at least one BES-predicted clone in at least one dimension. Two-dimensional success rates were consistently lower than either of the one-dimensional rates. This result is expected, as the failure of an overgo in either of the two dimensions would result in an overall false negative result. As seen in Figure 2, 60% of the tested overgos identified at least one BAC clone in both dimensions. Based on the average one-dimensional percentages, the maximum expected percentage of overgos that would detect at least one overgo in two dimensions is estimated to be (0.82 × 0.82 =) 67%. Therefore an estimated 7% of overgos overall were successful in one dimension but failed in the second dimension, although the chromosome 3 overgos had a 16% higher two-dimensional success rate than the chromosome 1 overgos.

Table 2 lists the success rates of overgos for all of the *Oryza *species tested, as well as the percentage of successful overgos that were designed from EST sequences in each case. Species are listed in the table in phylogenetic order, according to Ge et al. (1999). Not surprisingly, the greatest percentage of overgos were successful in *O. rufipogon*, an AA genome species like *O. sativa*. Hybridization success rates fell as species lower in the phylogenetic hierarchy were examined. Overgos hybridized least successfully to *O. brachyantha *(FF genome), *O. ridleyi *(HHJJ genome), and *O. granulata *(GG genome), the most basal genomes in the *Oryza *phylogeny. As hypothesized, the sequences from which the overgos were designed became more critical with increasing evolutionary distance from *O. sativa*. Approximately 50% of all overgos designed showed similarity to sequences in the EST databases. As shown in Table 2, 54% of the overgos that hybridized successfully to the *O. sativa *BAC library were designed from EST sequences. This is the expected result, since overgos designed from *O. sativa *should hybridize back to the *O. sativa *genome regardless of the character of the overgo sequence. Genic regions, from which EST sequences are derived, tend to be more conserved during evolution, so we hypothesized that overgos not designed from EST sequence would not work as well as EST-derived overgos as we moved farther down the phylogenetic order of species. We observed that as we tested species more evolutionarily distant from *O. sativa*, in particular non-AA genome species, fewer non-EST-derived overgos were successful in hybridizing to a particular library. In the species most distantly related to *O. sativa*, 80–83% of the successful overgos were derived from EST sequences.

**Table 2:** Overgo success rates in all *Oryza *species

Library^a^	Genome Type	No. Overgos Tested	% Successful Overgos	% Successful Overgos Designed From ESTs
*O. sativa*	AA	792	60	54
*O. rufipogon*	AA	432	56	43
*O. nivara*	AA	324	44	36
*O. punctata*	BB	540	31	82
*O. minuta*	BBCC	792	41	71
*O. officinalis*	CC	756	36	74
*O. alta*	CCDD	792	39	71
*O. australiensis*	EE	792	48	60
*O. coarctata*	HHKK	468	33	83
*O. brachyantha*	FF	792	15	82
*O. ridleyi*	HHJJ	468	28	80
*O. granulata*	GG	468	26	82

^a^Species are listed in phylogenetic order according to Ge et al. (1999) [11], with the species most closely related to *O. sativa *at the top of the table and the most distant relative at the bottom.

### Hybridization of overgos to *Sorghum*

**Figure 3:**
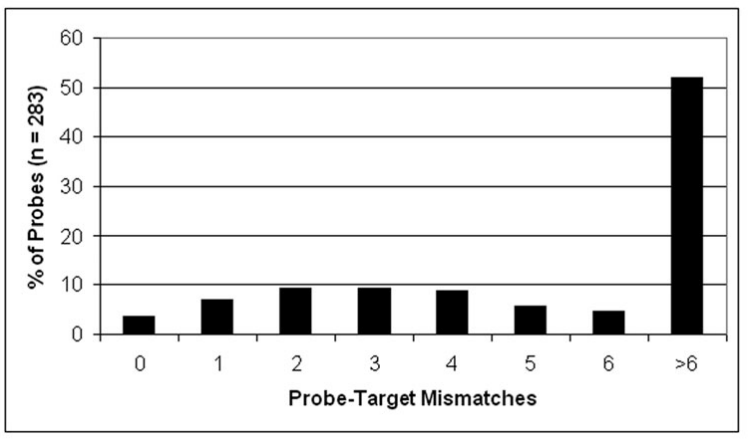
**Distribution of mismatches between rice overgos and *Sorghum *sequence**. The percentage of 36 bp overgo probes having 0 to >6 mismatches to available *Sorghum *sequence is shown. *Sorghum *sequences corresponding to regions of rice from which overgo probes were designed were identified using BLAT alignment data available from the Gramene (version 19) database [33]. Overgo sequences were aligned to *Sorghum *sequence by BLASTN using an open-gap cost of two and a gap-extension cost of one, with low-complexity filtration turned off.

To determine the ability of probes to detect sequences of a more distantly related member of the grass family we conducted a pilot study using *Sorghum bicolor*. Previously, Klein et al. (2003) [16] constructed an integrated genetic and physical map of sorghum and demonstrated extensive colinearity over the lengths of chromosome 3 and rice chromosome 1 [16]. For the current experiment, 3402 physically mapped sorghum BAC clones, spanning ~60 Mb of chromosome 3 (~10X coverage), were hybridized with 288 randomly selected overgo probes. Sixteen probes (5.6%) successfully identified at least one BAC clone, consistent with the trend of decreasing hybridization success rate with increasing evolutionary distance from rice. To better understand the relationship between hybridization success rate and sequence conservation we examined the level of probe-target mismatches in available sorghum sequences. Although available sequence of sorghum is limited, we were able to identify EST and genome survey sequences corresponding to regions of rice that encompass 283 of the 1721 probes, and these were subsequently examined by BLASTN to determine the level of mismatches. As shown in Figure 3, fewer than 4% of probes had zero mismatches, while approximately half had six or greater mismatches. We note that this sample of probes is biased toward greater conservation than the probe set as a whole since it excludes probes targeted to regions of rice for which no homologues exist in sorghum. Among probes for which we were able to obtain mismatch data, the hybridization success rate was 23% (9 out of 39). This was significantly higher (P < 0.001, chi-square test) than the success rate of probes for which no mismatch data was obtainable, which was 3% (7 out of 242). We further found that among the nine positively-hybridizing probes for which we have mismatch data, all but one had six or fewer mismatches, while the success rate among all 20 probes having six or fewer mismatches was 40%, compared to only 5.6% (1 out of 19) for probes having greater than 6 mismatches (data not shown).

### Comparative mapping of the rice and wild rice (*Oryza*) genomes

**Figure 4:**
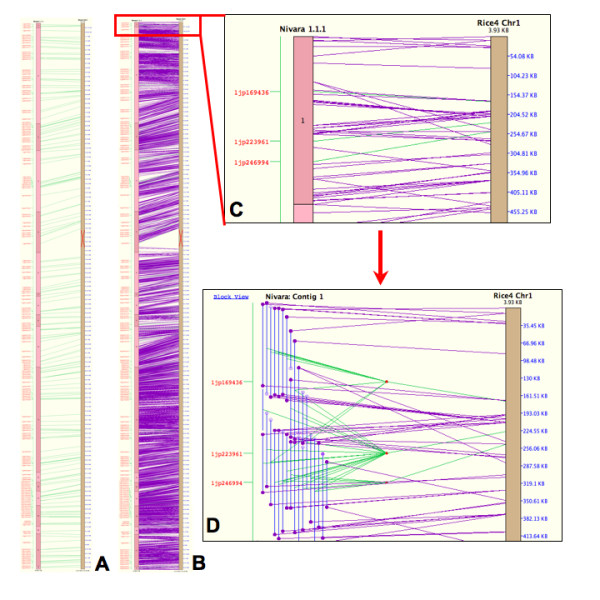
**Comparative physical map of *O. nivara *with rice chromosome 1**. SyMap screenshots showing the completed physical map of *O. nivara *chromosome 1 aligned to the *O. sativa *chromosome 1 pseudomolecule. (**A**) Whole-chromosome view of the *O. nivara *pseudomolecule aligned to the *O. sativa *chromosome 1 pseudomolecule, showing overgo marker alignments only. (**B**) Whole-chromosome view of the *O. nivara *pseudomolecule aligned to the *O. sativa *chromosome 1 pseudomolecule, showing both overgo marker and BAC end sequence (BES) alignments. (**C**) Zoomed-in view of the overgo and BES alignments between *O. nivara *contig 1 and the *O. sativa *chromosome 1 pseudomolecule. (**D**) More detailed view of (C) showing the actual clones comprising *O. nivara *contig 1 and their BESs. In this view, the alignments of individual BES can be seen, as well as individual clones that were detected by overgo markers, and the alignments of those markers to the *O. sativa *chromosome 1 pseudomolecule. In (A-C), BAC contigs are represented by numbered blocks which are stacked vertically to form the *O. nivara *pseudomolecule shown on the left of each alignment, while the *O. sativa *pseudomolecule is shown in brown on the right of each alignment. The red 'X' on the *O. sativa *pseudomolecule represents the centromere. Overgo marker names are listed in red text to the left of each alignment, while coordinates along the *O. sativa *pseudomolecule are listed in blue text to the right of each alignment. Green lines stretching from the *O. nivara *pseudomolecule to the *O. sativa *pseudomolecule in each alignment show where clones from the *O. nivara *contig align to the *O. sativa *chromosome, while purple lines show where *O. nivara *clones' BESs align to the *O. sativa *pseudomolecule. In (D), blue vertical lines on the left half of the figure represent *O. nivara *BAC clones. Circles on the ends of the clones represent BESs. Open circles are BESs that did not match sequences from *O. sativa*, while closed circles are BESs that matched *O. sativa *sequences. Purple lines stretching from a BES on the left to the pseudomolecule on the right show where the BES to which the line is attached aligns to the pseudomolecule. In the case of overgo markers, a marker will often hit more than one BAC clone. Green lines stretch from the middle of all clones hit by that marker to a red "marker join dot." The green line stretching from the marker join dot to the pseudomolecule shows where the marker sequence is located on the pseudomolecule, thereby showing where the *O. nivara *clones hit by the marker align to the *O. sativa *pseudomolecule.

**Figure 5:**
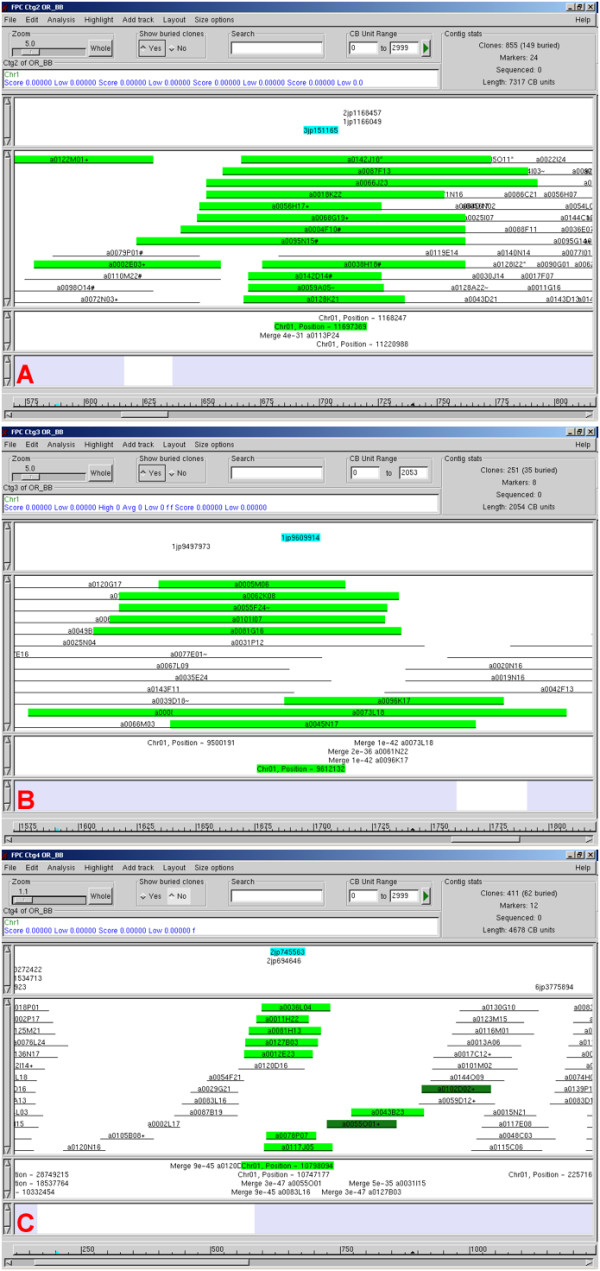
**Contig merges driven by overgos in *O. nivara ***. Panels A-C display three examples from FPC of contig merges driven by overgo hybridizations. Overgo names are highlighted in blue, while BAC clones to which those overgos hybridized are highlighted in green.

Clone data from the overgo probe hybridizations, in conjunction with clone fingerprint data, was used to construct physical maps of the rice chromosome 1 orthologues in *O. nivara *and *O. officinalis*. The *O. nivara *physical map of chromosome 1 is shown in Figure 4. While in most cases it is not possible to ascertain whether overgo markers were solely responsible for contig builds or merges in the physical map, we have determined that at least 89 of the overgo markers shown in Figure 4 confirmed the *in silico *alignment of the BES to the corresponding pseudomolecule of *O. sativa *chromosome 1, providing robustness to the physical map. Close inspection of the physical maps also revealed areas where overgos aided in the assembly of contigs, especially by bringing together clones in areas of low coverage. Three examples of these areas are shown in Figure 5.

**Figure 6:**
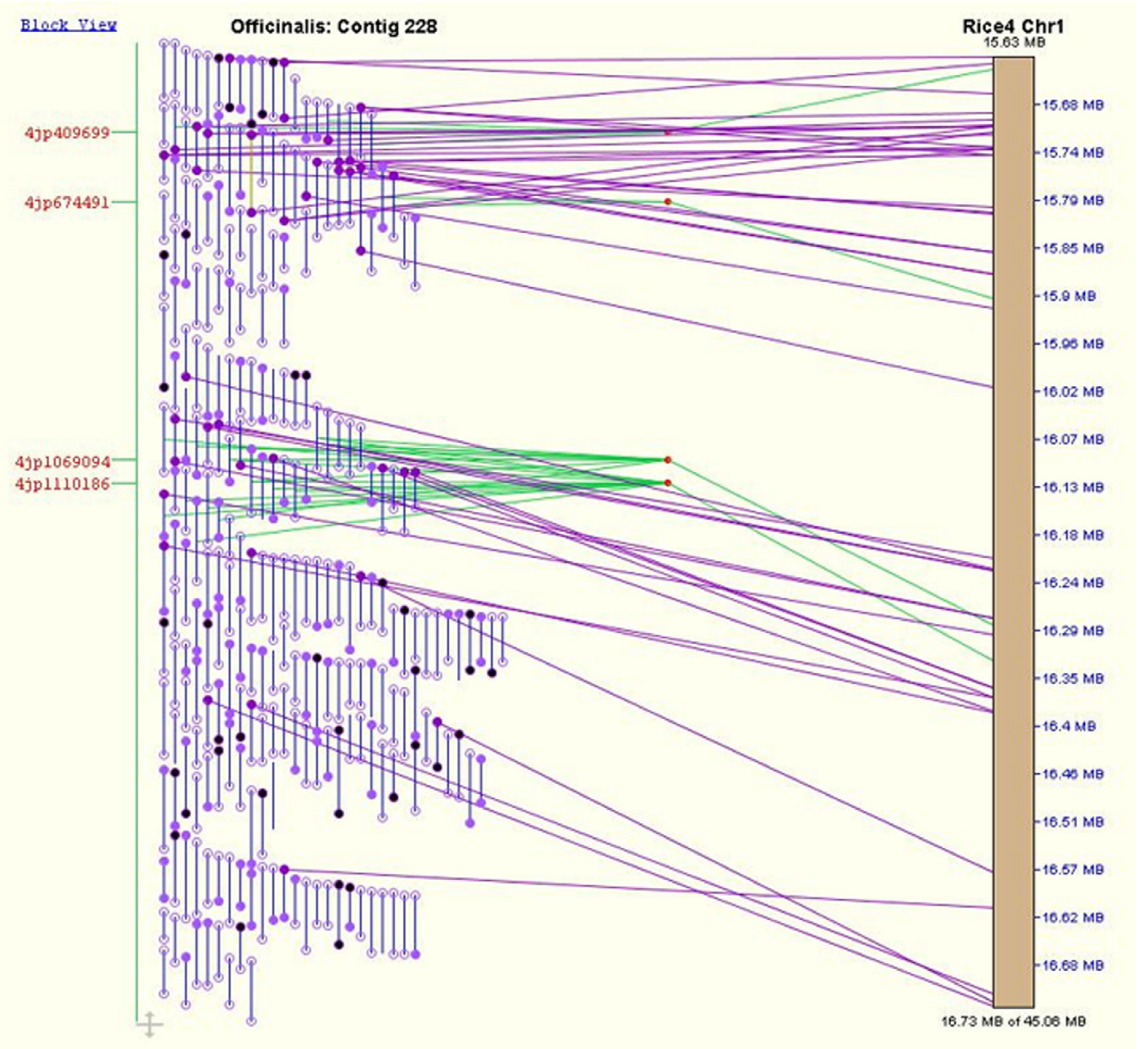
**Alignment of *O. officinalis *contig to rice chromosome 1**. A detailed view of the alignment of an *O. officinalis *BAC contig to the *O. sativa *chromosome 1 pseudomolecule using BAC end sequences (BES) and overgo markers. The hybridizations of overgos 4jp1069094 and 4jp1110186 in particular to *O. officinalis *BAC clones drove the merger of two initially separated contigs to form the *O. officinalis *contig shown here. The brown bar on the right side of the figure represents a portion of the *O. sativa *chromosome 1 pseudomolecule, and the coordinates along the pseudomolecule are listed in blue on the righthand side of the pseudomolecule. Blue vertical lines on the left half of the figure represent *O. officinalis *BAC clones. Circles on the ends of the clones represent BESs. Open circles are BESs that did not match sequences from *O. sativa*, while closed circles are BESs that matched *O. sativa *sequences. Purple lines stretching from a BES on the left to the pseudomolecule on the right show where the BES to which the line is attached aligns to the pseudomolecule. Red text on the left side of the figure shows the names of overgo markers with hits to clones in the *O. officinalis *BAC contig shown. In the case of overgo markers, a marker will often hit more than one BAC clone. Green lines stretch from the middle of all clones hit by that marker to a red "marker join dot." The green line stretching from the marker join dot to the pseudomolecule shows where the marker sequence is located on the pseudomolecule, thereby showing where the *O. officinalis *clones hit by the marker align to the *O. sativa *pseudomolecule.

**Figure 7:**
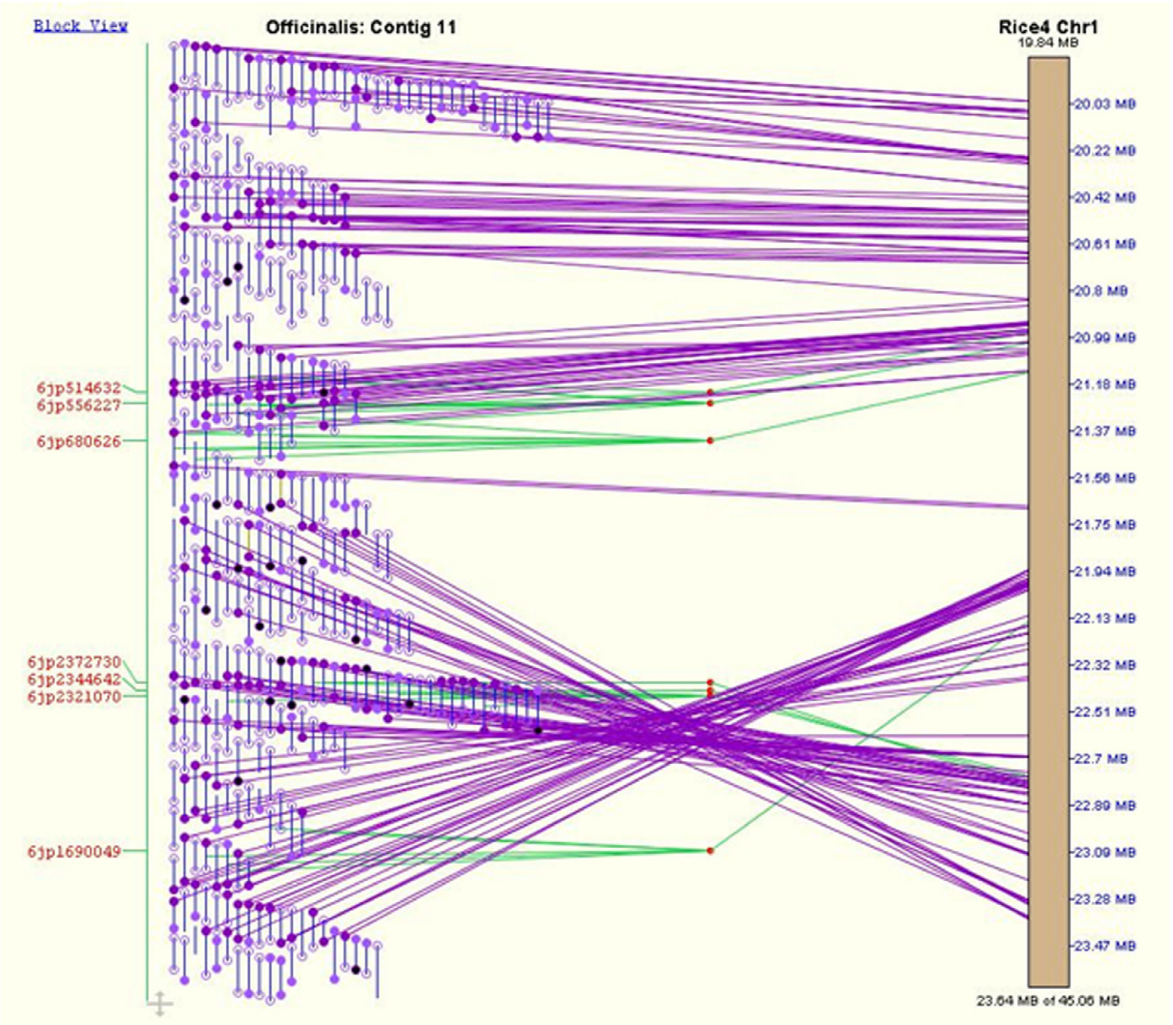
**Alignment of *O. officinalis *contig to region of rice chromosome 1 showing putative inversion**. Alignment of an *O. officinalis *contig to rice chromosome 1. Overgos confirm the placement of clones in the contig such that a putative genomic inversion of the region stretching from approximately 21.94 MB to 23.40 MB on the rice pseudomolecule is apparent. The brown bar on the right side of the figure represents a portion of the *O. sativa *chromosome 1 pseudomolecule, and the coordinates along the pseudomolecule are listed in blue on the righthand side of the pseudomolecule. Blue vertical lines on the left half of the figure represent *O. officinalis *BAC clones. Circles on the ends of the clones represent BAC end sequences (BES). Open circles are BESs that did not match sequences from *O. sativa*, while closed circles are BESs that matched *O. sativa *sequences. Purple lines stretching from a BES on the left to the pseudomolecule on the right show where the BES to which the line is attached aligns to the pseudomolecule. Red text on the left side of the figure shows the names of overgo markers with hits to clones in the *O. officinalis *BAC contig shown. In the case of overgo markers, a marker will often hit more than one BAC clone. Green lines stretch from the middle of all clones hit by that marker to a red "marker join dot." The green line stretching from the marker join dot to the pseudomolecule shows where the marker sequence is located on the pseudomolecule, thereby showing where the *O. officinalis *clones hit by the marker align to the *O. sativa *pseudomolecule.

Figure 6 illustrates a contig of the *O. officinalis *genome that was originally assigned to rice chromosome 11 based on the alignment of BES data from an FPC contig to the rice sequence, although repeats and duplications in the region caused this to be characterized as an ambiguous assignment. Four overgo markers resolved the ambiguity, allowing the contig to be placed unambiguously on the rice chromosome 1 comparative map. Overgos have also confirmed the placement of contigs around at least one region of the *O. officinalis *genome containing a chromosomal rearrangement. This is shown in Figure 7, where two overgos support an inversion in the comparative map of *O. officinalis *with rice chromosome 1. Three additional overgos upstream of this rearrangement point to colinearity of the chromosome outside of the putative inversion breakpoints.

**Figure 8:**
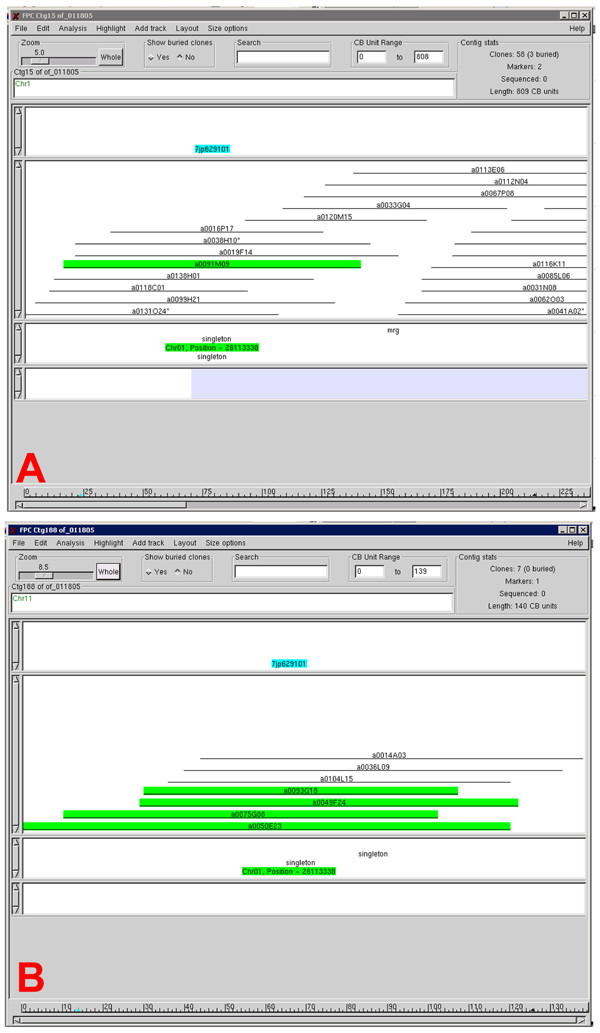
**Merger of two *O. officinalis *contigs by a single overgo probe**. FPC views of contigs 15 (A) and 188 (B) of *O. officinalis*. These two contigs were merged in the physical map based on the hybridization of overgo 7jp629101 to both contigs, leading to an additional merger with contig 16 (not shown).

The assembly and alignment of physical contigs from a wild rice species to the reference genome is often made more difficult by lower degrees of synteny between the two species in these areas, as well as by the presence of repetitive elements and rearrangements. In these cases, overgo markers confirmed orthologous tracks in the chromosomes of the wild species in reference to *O. sativa*. One example of this can be seen in *O. officinalis*. Contig 15, shown in Figure 8A, was merged with *O. officinalis *contig 188 (Figure 8B), based on the hybridization of overgo 7jp629101 to BAC clones from both contigs. After this merger, fingerprint data then pointed to a merger of contig 16 with contig 15/188, a merger that could not be made from fingerprint data alone, but which was driven by overgo 7jp629101 (data not shown). In the process, the data from the same overgo resolved a false inversion that had been observed in contig 15 prior to the merger.

### Comparative mapping of the rice and *Sorghum *genomes

**Figure 9:**
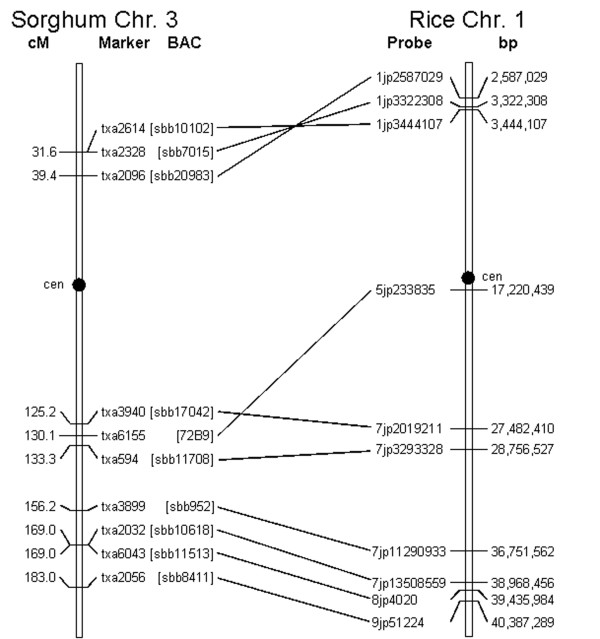
**Comparative map between *Sorghum *chromosome 3 and rice chromosome 1**. Hybridization of overgo probes detected the indicated BAC clones, which were previously anchored to the *Sorghum *genetic map using a variety of molecular markers [16, 17]. The rice physical map is based on the TIGR Release 3 pseudochromosome assembly [39]. The data show a previously identified inversion event affecting the short arms of the chromosomes. Probe 5jp233835 identified a locus that has possibly moved in one lineage relative to the other.

Based on the probe-to-BAC hybridization data and the integrated genetic and physical maps of sorghum [16,17], we drew a comparative map of sorghum chromosome 3 and rice chromosome 1. Shown in Figure 9, the map displays long-range colinearity between the two chromosomes, with the exception of one locus showing evidence of having relocated in one lineage relative to the other. In addition, the map detects an inversion event that was previously shown to encompass most of the short arms of each chromosome [16]. These results are in agreement with previous findings of a syntenic relationship between these chromosomes, with small-scale changes resulting from movement of individual or small clusters of genes [16,18].

## Discussion

Overgo probes have been gaining popularity as markers for large-scale physical mapping of both plant and animal genomes. They are particularly useful for screening large-insert libraries to identify clones belonging to regions of interest, or to identify clones to sequence and fingerprint for contig assembly [19,20]. One disadvantage of overgos is that good quality sequence information must be available from which to design the probe. In addition, repeat databases that can be used to filter out repetitive probes are also essential. On the other hand, overgo probes can be designed from sequences that are too short for optimal PCR primer selection when a hybridization probe from the region is needed. Overgo probes can also be labelled to a high specific activity and can be pooled easily, making them ideal candidates for high-throughput physical mapping [19].

The criteria used to select overgos for hybridization experiments play a huge role in the success of these markers. Most of the studies reported to date in both plants and animals have used overgos designed from the sequence of one species to probe clone libraries of that same species [4,21-30]. In animals, where comprehensive sequence data is available for many species, overgos have been designed from the conserved regions of aligned sequences from multiple species [9,10,31,32]. The overgo design process for our rice comparative mapping project presented a unique problem. As evidenced in the studies cited above, it is necessary to design overgos as much as possible to conserved regions for successful cross-species hybridizations, yet the plant community does not have the large repository of complete sequence data that exists for animal and microbial species. At the time this study began, *Arabidopsis thaliana *(L.) Heynh. was the only plant species that had been sequenced to completion, although rice chromosome 1 had been completed and the rest of the rice sequence was soon published [2,3]. While a multispecies alignment approach to overgo selection is preferable, these resources were not available, so we selected overgos with a bias towards hits to available plant EST databases.

The success rate of overgos in this study was 82%, a rate that is comparable to the rates reported by other studies [21-23,27,29,31]. Our two-dimensional overgo success rate also closely matches the rate we would expect based on the overall success of overgos in one dimension. Additionally, these rates are supported by the fact that overgos not only had to positively hybridize to a clone, but that the clone must map to the expected location in the FPC and BES maps, criteria not used by prior studies using overgos. The two-dimensional success rate could be improved in future experiments by the addition of additional dimensions, possibly coupled with an increase in the number of overgos per pool. Using three dimensions and requiring a positive result in two of the three dimensions, for example, would increase the success rate by reducing the number of false negatives due to the failure of an overgo in one of the dimensions. In addition, our data point to a need for the continuous improvement of available sequence databases within the plant community, particularly EST databases; the continued sequencing of other genomes that could be aligned to rice to disclose regions of sequence conservation will also contribute to improved overgo design and an increase in probe success. We have shown that this is particularly important for cross-species hybridizations. For example, while only 26% of the overgos successfully hybridized to *O. coarctata *BAC clones, 83% of the overgos that were successful were designed from EST sequences. This fact was also shown by our *Sorghum *data, in which 40% of rice overgos with six or fewer mismatches to *Sorghum *yielded positive hybridization results, compared to only 5.6% of overgos with greater than six mismatches. The improvement of repeat databases to screen out overgos designed from repetitive regions will also be crucial to future cross-species overgo success.

Even given the current overgo design constraints, this study shows that overgos can be used successfully as markers for the construction of physical maps, as evidenced by our data from *O. nivara *and *O. officinalis*. Besides confirming the placement of clones into contigs using fingerprint data, we have shown here the use of these overgo markers to resolve conflicts in the chromosomal assignment of contigs and to drive the merges of contigs during physical map construction. In addition, we have shown evidence that overgos can be used successfully to confirm the placement of contigs in and around regions containing suspected rearrangements in comparative maps, a possibility that will be examined in greater detail in future investigations. As evolutionary distance increases relative to the reference genome, it becomes more difficult to design overgos that will be able to successfully hybridize to the genomes of other species. Yet even in *Sorghum*, a species outside of the genus *Oryza*, overgo probes were successfully used in this study to aid in the construction of a comparative map between rice and *Sorghum*, and were able to show micro-synteny between regions of the *Sorghum *and rice genomes.

## Conclusion

Overgos have been used successfully for physical mapping in animals, but their utility for physical and comparative mapping in plants has not been studied as extensively as in animals. The study presented here shows that overgos can be successfully hybridized cross-species, and can even be used with limited success in species as distantly related as rice and *Sorghum*. Of particular interest will be the possibility of using overgo probes to target potential regions of chromosomal rearrangements and to target gaps in the physical maps to drive the merging of contigs and the placement of singletons in those maps. The improvement of sequence databases, including EST databases, and repeat databases will be instrumental in the successful design of these probes for cross-species hybridizations.

## Methods

### Overgo design

Overgo probes consisted of 36 bp stretches of sequence chosen from the Rice Genome Research Program's publicly available sequence data of the nine contigs of *Oryza sativa *japonica L. cv. Nipponbare, chromosome 1 [14]. Each overgo is composed of two 22 mer oligonucleotides that overlap at their 3' ends via 8 bp of complimentary sequence. A collection of candidate overgo sequences were extracted from each contig using the program SOOP [9], which was modified to display all potential overgos found in a sequence, instead of only the best overgo for a given sequence. Candidate overgos were discarded unless they were composed of 44–56% GC bases and displayed less than 24/36 bases (32/36 bases for the pilot overgo set, plate 21) of sequence identity to other sites on the chromosome from which they were extracted. The remaining candidate overgos were then scored against publicly available databases [33] as follows. For chromosome 1 candidate overgos, a score of +5 was given for hits to the grain EST database (excluding Japonica and Indica); +3 for hits to the Indica EST database; +3 for hits to the Japonica chromosome 1 EST database; +1 for hits to the Japonica EST database; -1 for 4 to 7 hits, or -5 for 8 or more hits, to the Indica genome database; and -2 for 4 to 7 hits, or -5 for 8 or more hits, to the Japonica genome database. For chromosome 3 candidate overgos, a score of +5 was given for hits to the grain EST database (excluding Japonica and Indica); +3 for hits to the Indica EST database; +1 for hits to the Japonica EST database; and -1 for 4 to 7 hits, or -5 for 8 or more hits, to the Indica genome database. Overgos were given a score of zero if they hit more than once to the Japonica genome database.

Overgos suitable for use as hybridization probes were selected to have the best possible score (minimum score of 8) while achieving an optimum density of one overgo every 25,000 bp. The overgos were ordered in 96-well plates from Sigma-Genosys, maximizing the distance between overgos in a 12 × 12 pooling array to a distance equalling or exceeding the maximum BAC clone insert size. Chromosome 1 overgos had an average of 3,610,538 bp between any two overgos in a row pool and 4,243,653 bp between any two overgos in a column pool, while the chromosome 3 overgos had an average spacing of 2,340,440 bp between any two overgos in a row pool and 1,498,379 bp between any two overgos in a column pool. All overgo sequences, each overgo's position on the chromosome from which it was designed, the overgo's score, and the number of times the overgo matches each of the databases, can be found at [34]. A list of the sequences and plate positions of all 864 overgos used in this study is provided [see Additional file 1].

### BAC libraries

The following 10x or greater coverage BAC libraries were obtained from the Arizona Genomics Institute [35]: OSJNBa (*Oryza sativa *L. ssp. *japonica *cv. Nipponbare; AA genome), OA_ABa (*Oryza australiensis *Domin.; accession 100882; EE genome), OA_BBa (*Oryza alta *Swallen; accession 105143; CCDD genome), OB__Ba (*Oryza brachyantha *A. Chev. et Roehr.; accession 101232; FF genome), OC__Ba (*Oryza coarctata *Roxb.; accession 104502; also known as *Porteresia coarctata *T.; HHKK genome), OG_ABa (*Oryza granulata *Nees et Arn. ex Watt; accession 102118; GG genome), OM__Ba (*Oryza minuta *J.S. Presl. ex C.B. Presl.; accession 101141; BBCC genome), OO__Ba (*Oryza officinalis *Wall ex Watt; accession 100896; CC genome), OP__Ba (*Oryza punctata *Kotschy ex Steud.; accession 105690; BB genome), OR_ABa (*Oryza ridleyi *Hook.; accession 100821; HHJJ genome), OR_BBa (*Oryza nivara *Sharma et Shastry; accession W0106; AA genome), and OR_CBa (*Oryza rufipogon *Griff.; accession 105491; AA genome).

One species of note that was included in our study is *Oryza coarctata *Roxb., also known as *Porteresia coarctata *T. Taxonomists in the 1980s moved the *coarctata *species out of the genus *Oryza *and into the genus *Porteresia*. Recently, Ge et al. (1999) [11] found this species to contain an HHKK genome type, like that of *Oryza schlechteri *Pilger, and suggested that the *coarctata *species be placed back into the genus *Oryza*. For that reason, we chose to include the *coarctata *species in our study of *Oryza *wild rice species.

Overgos designed from chromosome 1 were hybridized to the following BAC libraries: OSJNBa, OA_ABa, OA_BBa, OB__Ba, OC__Ba, OG_ABa, OM__Ba, OO__Ba, OP__Ba, OR_ABa, OR_BBa, and OR_CBa. The overgos designed from chromosome 3 were tested against all of the libraries listed above except for OR_CBa. While OSJNBa, OA_ABa, OA_BBa, and OB__Ba were hybridized against all of the overgos, the other libraries were incorporated into the study as they became available and were therefore tested against only portions of the overgos from those arrays.

### Filter printing

High-density nylon filter arrays of each library were fabricated as follows. 22 × 22-cm Hybond N+ nylon filters (Amersham GE Healthcare cat. no. RPN2222B) were placed on the surface of 250 mL of agar-solidified LB media supplemented with 10 mg/ml chloramphenicol (Sigma cat. no. C-0378) in Qtrays (Genetix cat. no. x6023). A Total Array System robot (BioRobotics) fitted with a 0.4 mm 384-pin tool double spotted the contents of 48 glycerol stock plates (18,432 clones) on each nylon filter. Qtrays holding the filters were incubated overnight at 37°C.

The resulting colony filters were fixed by treatment with a series of four solutions in the following manner. In preparation for fixing, Cellulose Chromatography Thick paper (size 46 × 57 cm; Fisher Scientific cat. no. 05-714-4) was cut and placed on 26 × 18 inch fiberglass cafeteria trays (Carlisle cat. no. 2618SL). The chromatography paper was moistened thoroughly with solution and excess solution was poured off. Nylon filters were placed on the solution-moistened paper for a specified period of time and then either moved to the next solution or dried, as detailed below. Filters were dried at room temperature by peeling the filter from moistened paper after incubation and placing it directly on the surface of an empty fiberglass tray. The fixing regimen proceeded according to the following steps. Filters were peeled from the surface of the agar-solidified media, treated for 7 minutes with 0.5 N NaOH and 1.5 M NaCl, then treated for 7 minutes with 1.5 M NaCl and 0.5 M Tris, (pH 8.0 at 25°C). Next, the filters were dried for more than one hour, then treated for 20 minutes with 0.4 N NaOH, followed by treatment for 7 minutes with 4X SSPE, pH 7.4. Following these incubations, the filters were allowed to dry overnight. Finally, each filter was UV cross-linked using a Stratalinker 2400 (Stratagene) set on ''Auto'' to deliver 120 joules/cm^2 ^to each filter.

Each filter was labelled by hand with the library/filter designation (e.g. OR_BBa A) and a unique serial number using a black Sanford Uni-Ball fine-point pen. A 1/16" diameter hole punch (Making Memories) was then used to punch holes in the filter at the corners of each of the six panels. Filters generally had a gray background on phosphorimager scans or autoradiographs. The holes in the filter appeared as white spots in an otherwise gray background and were used as guides to determine hit coordinates in ComboScreen (see Data Analysis below).

### Overgo labelling and hybridization

Forward and reverse oligonucleotides for each overgo were obtained from Sigma-Genosys at a stock concentration of 100 μM. Overgo probes were labelled and BAC filters hybridized using protocols previously described [19,20,31], with minor modifications. Forward and reverse oligonucleotides for each overgo were combined to a working concentration of 0.2 pmol/μL. For each labelling reaction, 4.5 μL of this working solution was incubated at 80°C for 5 minutes, followed by 37°C for 10 minutes, to anneal the 3' ends of the oligonucleotides. To the annealed overgos for each reaction, 1.6 μL OLB [20], 4 μg Bovine Serum Albumin (New England Biolabs cat. no. B9001S), 4 μCi [α^32^P]dCTP (3000 Ci/mmol; Amersham Biosciences cat. no. AA0005), 4 μCi [α^32^P]dATP (3000 Ci/mmol; Amersham Biosciences cat. no. AA0004), 0.8 units DNA Polymerase I Large (Klenow) Fragment (New England Biolabs cat. no. M0210L), and water were added to a final reaction volume of 8 μL. The reactions were incubated at room temperature for 1.5 hours, then combined into pools of twelve probes each and purified through NICK columns (Amersham Biosciences cat. no. 17-0855-02), eluting the probe in 400 μL TE buffer (pH 8.0).

BAC filters were soaked in 2X SSC [36], then prehybridized in 50 mL Church Buffer [36] for 30 minutes at 58°C. Probes were denatured at 95°C for 5 minutes, chilled on ice for 2 minutes, then 80 μL of probe was added to each hybridization bottle and the filters were hybridized at 58°C overnight. Filters were washed in 1.5X SSC and 0.1% SDS at 58°C for 30 minutes and then in 1.0X SSC and 0.1% SDS at 58°C for 30 minutes. Filters were exposed to 23 × 25 cm type MS imaging plates (FUJIFILM Medical Systems USA, Inc., cat. no. YBIP2325MS) for at least 17 hours, then the imaging plates were scanned using the FLA-5000 imaging system with Image Reader FLA-5000 v2.1 and ImageGauge 4.0 software (FUJIFILM Medical Systems USA, Inc.) at 16 bit gradation and a resolution of 200 μm. Images were cropped, labelled, and contrast adjusted using Adobe^® ^Photoshop^® ^CS software.

### Data analysis

A two-dimensional pooling strategy was used to identify the specific probe hybridizing to a specific clone while reducing the total number of hybridizations needed to identify all positive clones with all overgos. Overgos were pooled in 12-overgo pools by row and by column, and row and column pools were used to probe the BAC filters. Images from the hybridizations were scored and the data assimilated into a database format using the program ComboScreen [37,38]. ComboScreen was modified to include the following features: image rotation, adjustment of image brightness and contrast, ability to rotate the grid, ability to copy a sized grid from one panel to another, sorting of the hybridization bottles list, recall of scoring information (grid information saved as .gda file), and the ability to score the intensity of the hybridization signal. Data were analyzed using custom programs designed to display data in a web-based format [34].

### Anchoring BAC clones to rice sequence

BAC clones from the OSJNBa (*O. sativa*) library were anchored to *O. sativa *chromosome sequences (Release 2) constructed by The Institute For Genomic Research (TIGR) [39] from completed BAC sequences produced by the International Rice Genome Sequencing Project (IRGSP) [40] using both BAC end sequences (BES) of OSJNBa clones and fingerprint (FPC) data of the same clones. BES [41] and FPC [42] data files were obtained from the Arizona Genomics Institute and Computational Laboratory (AGI). BLAT [43] was used to generate position data for BES forward and reverse reads on the chromosome sequences. BAC clones were excluded from further analysis unless both forward and reverse reads were uniquely positioned and within 300 kb of each other.

To anchor BAC clones to the *O. sativa *chromosome sequence using FPC data, FPC files were converted into GFF format using a PERL script [44]. The GFF file contains size and overlap estimates of the FPC clones. Use of these estimates resulted in smaller chromosome lengths than those obtained from the chromosome sequences Therefore, manual resizing of the FPC-based map was undertaken as follows. First, clones on either side of the FPC clone in question were analyzed to find the nearest flanking clones that were both found in the FPC map and sequenced and positioned on the chromosome sequence. Once left and right flanking clones were found meeting those criteria, the clones' sizes and positions in both FPC and on the chromosome sequence were compared. The comparisons were then used to stretch or shrink the FPC clone, then position it between the two sequenced clones on the chromosome sequence, retaining the original aspect ratio to maintain relative clone size and position.

## Authors' contributions

BLH-J was the project manager for the overgo hybridization work, designed the overgo hybridization experiments, participated in and supervised the scanning and scoring of BAC filters, analyzed data, and drafted the manuscript. MF-G carried out overgo hybridization experiments, including scanning and scoring BAC filters, and critiqued the manuscript for intellectual content. BA wrote scripts for the project, maintained the databases of found BAC clones, and participated in data analysis. RW designed all overgos used in the study. J-LG did the manual editing of all but one of the FPC physical maps, participated in the data analysis, and edited drafts of the manuscript. JS and BH performed the comparative mapping analysis between *Sorghum *and rice using the overgo hybridization data. JS also edited drafts of the manuscript. PK and BZ constructed the *Sorghum *chromosome 3 tiling path.. FR printed all of the BAC library filters used in this project. AS, NG, J-YL, and JGW carried out overgo hybridization experiments, including scanning and scoring BAC filters. MZL made the DNA plugs from which all of the BAC libraries were built. JSSA constructed the BAC libraries used in this study. DK created the initial FPC physical map builds. HRK did the manual editing of the *O. punctata *FPC build. DW conceived of and coordinated the rice-*Sorghum *comparative mapping study, and was its principal investigator. RAW is the principal investigator on the grant that funded the BAC library construction and the FPC physical mapping. PSM and SAJ contributed significantly to the design of the rice experiments described in this paper, contributed to the analysis and interpretation of data, and critiqued the manuscript for intellectual content. All authors read and approved the final manuscript.

## Supplementary Material

Additional File 1 Overgo plate position and sequence information. Click here for file
